# Field methods for sampling tree height for tropical forest biomass estimation

**DOI:** 10.1111/2041-210X.12962

**Published:** 2018-02-13

**Authors:** Martin J. P. Sullivan, Simon L. Lewis, Wannes Hubau, Lan Qie, Timothy R. Baker, Lindsay F. Banin, Jerôme Chave, Aida Cuni‐Sanchez, Ted R. Feldpausch, Gabriela Lopez‐Gonzalez, Eric Arets, Peter Ashton, Jean‐François Bastin, Nicholas J. Berry, Jan Bogaert, Rene Boot, Francis Q. Brearley, Roel Brienen, David F. R. P. Burslem, Charles de Canniere, Markéta Chudomelová, Martin Dančák, Corneille Ewango, Radim Hédl, Jon Lloyd, Jean‐Remy Makana, Yadvinder Malhi, Beatriz S. Marimon, Ben Hur Marimon Junior, Faizah Metali, Sam Moore, Laszlo Nagy, Percy Nuñez Vargas, Colin A. Pendry, Hirma Ramírez‐Angulo, Jan Reitsma, Ervan Rutishauser, Kamariah Abu Salim, Bonaventure Sonké, Rahayu S. Sukri, Terry Sunderland, Martin Svátek, Peter M. Umunay, Rodolfo Vasquez Martinez, Ronald R. E. Vernimmen, Emilio Vilanova Torre, Jason Vleminckx, Vincent Vos, Oliver L. Phillips

**Affiliations:** ^1^ School of Geography University of Leeds Leeds UK; ^2^ Department of Geography University College London London UK; ^3^ Laboratory for Wood Biology and Xylarium Royal Museum for Central Africa Tervuren Belgium; ^4^ Department of Life Sciences Imperial College London Ascot UK; ^5^ Centre for Ecology and Hydrology Penicuik UK; ^6^ Université Paul Sabatier CNRS UMR 5174 Evolution et Diversité Biologique Toulouse France; ^7^ Environment Department University of York Heslington York UK; ^8^ Geography College of Life and Environmental Sciences University of Exeter Exeter UK; ^9^ Wageningen Envrionmental Research (Alterra) Wageningen University and Research Wageningen The Netherlands; ^10^ Department of Organismic and Evolutionary Biology Harvard University Cambridge MA USA; ^11^ Department of Environmental Systems Science Institute of Integrative Biology ETH Zürich Zürich Switzerland; ^12^ The Landscapes and Livelihoods Partnership Edinburgh UK; ^13^ Biodiversity and Landscape Unit Gembloux Agro‐Bio Tech Université de Liège Gembloux Belgium; ^14^ Institute for Environmental Biology Utrecht University Utrecht The Netherlands; ^15^ Tropenbos International Wageningen The Netherlands; ^16^ School of Science and the Environment Manchester Metropolitan University Manchester UK; ^17^ School of Biological Sciences University of Aberdeen Aberdeen UK; ^18^ Landscape Ecology and Plant Production Systems Unit Université Libre de Bruxelles Bruxelles Belgium; ^19^ The Czech Academy of Sciences Institute of Botany Brno Czech Republic; ^20^ Department of Ecology & Environmental Sciences Faculty of Science Palacký University Olomouc Czech Republic; ^21^ Faculty of Science Université de Kisangani Kisangani Democratic Republic of Congo; ^22^ Department of Botany Faculty of Science Palacký University in Olomouc Czech Republic; ^23^ School of Marine and Environmental Sciences James Cook University Cairns Qld Australia; ^24^ Faculdade de Filosofia Cîencias e Letras de Ribeirão Preto Universidade de São Paulo Ribeirão Preto Brazil; ^25^ Environmental Change Institute School of Geography and the Environment University of Oxford Oxford UK; ^26^ Universidade do Estado de Mato Grosso Nova Xavantina Brazil; ^27^ Environmental and Life Sciences Programme Faculty of Science Universiti Brunei Darussalam Brunei‐Muara Brunei Darussalam; ^28^ Universidade Estadual de Campinas Campinas Brazil; ^29^ Universidad Nacional de San Antonio Abad del Cusco Cusco Perú; ^30^ Royal Botanic Garden Edinburgh Edinburgh UK; ^31^ Instituto de Investigaciones para el Desarrollo Forestal Universidad de Los Andes Avenida Principal Chorros de Milla Campus Universitario Forestal Edificio Principal Mérida Venezuela; ^32^ Bureau Waardenburg bv Culemborg The Netherlands; ^33^ Carboforexpert Geneva Switzerland; ^34^ Smithsonian Tropical Research Institute Balboa, Ancon Panama; ^35^ Plant Systematic and Ecology Laboratory Department of Biology Higher Teachers’ Training College University of Yaounde I Yaounde Cameroon; ^36^ Center for International Forestry Research Bogor Indonesia; ^37^ Faculty of Forestry University of British Columbia Vancouver Canada; ^38^ Department of Forest Botany, Dendrology and Geobiocoenology Faculty of Forestry and Wood Technology Mendel University in Brno Brno Czech Republic; ^39^ Yale School of Forestry & Environmental Studies New Haven CT USA; ^40^ Jardín Botánico de Missouri Oxapampa, Pasco Perú; ^41^ Deltares Delft The Netherlands; ^42^ Department of Biological Sciences Florida International University Miami FL USA; ^43^ Universidad Autónoma del Beni Riberalta Bolivia; ^44^ Bolivia Centro de Investigación y Promoción del Campesinado ‐ Norte Amazónico Riberalta Bolivia

**Keywords:** above‐ground biomass estimation, allometry, carbon stocks, forest inventory, forest structure, sample size

## Abstract

1. Quantifying the relationship between tree diameter and height is a key component of efforts to estimate biomass and carbon stocks in tropical forests. Although substantial site‐to‐site variation in height–diameter allometries has been documented, the time consuming nature of measuring all tree heights in an inventory plot means that most studies do not include height, or else use generic pan‐tropical or regional allometric equations to estimate height.
2. Using a pan‐tropical dataset of 73 plots where at least 150 trees had in‐field ground‐based height measurements, we examined how the number of trees sampled affects the performance of locally derived height–diameter allometries, and evaluated the performance of different methods for sampling trees for height measurement.
3. Using cross‐validation, we found that allometries constructed with just 20 locally measured values could often predict tree height with lower error than regional or climate‐based allometries (mean reduction in prediction error = 0.46 m). The predictive performance of locally derived allometries improved with sample size, but with diminishing returns in performance gains when more than 40 trees were sampled. Estimates of stand‐level biomass produced using local allometries to estimate tree height show no over‐ or under‐estimation bias when compared with biomass estimates using field measured heights. We evaluated five strategies to sample trees for height measurement, and found that sampling strategies that included measuring the heights of the ten largest diameter trees in a plot outperformed (in terms of resulting in local height–diameter models with low height prediction error) entirely random or diameter size‐class stratified approaches.
4. Our results indicate that even limited sampling of heights can be used to refine height–diameter allometries. We recommend aiming for a conservative threshold of sampling 50 trees per location for height measurement, and including the ten trees with the largest diameter in this sample.

## 1 INTRODUCTION

Tropical forests play a key role in the global carbon cycle and are a major carbon pool, with ca. 285 Pg of carbon estimated to be stored in above‐ground live biomass (Feldpausch et al., 2012). Current efforts to quantify global carbon stocks (e.g. Avitabile et al., 2016), understand carbon dynamics in tropical forests (e.g. Brienen et al., 2015), evaluate the potential for forest conservation to mitigate climate change (e.g. Jantz, Goetz, & Laporte, 2014) and examine biodiversity‐ecosystem function relationships (e.g. Chisholm et al., 2013) all rely on robust estimates of carbon storage in above‐ground biomass (AGB). The AGB of forests can be estimated from ground‐based inventory plots, where allometric equations are used to estimate AGB from measured tree diameters (Chave et al., 2014). Tree height is an important component of this allometric relationship, as tree biomass is partially a function of tree volume, which is, in turn, a function of tree height, trunk basal area and trunk taper (Chave et al., 2005). Incorporating a height parameter is known to markedly improve estimates of individual tree AGB (Feldpausch et al., 2012), and this has a substantial effect at larger scales too. For example estimates of global tropical forest biomass carbon stocks vary by 35.2 Pg depending simply on whether height is incorporated (Feldpausch et al., 2012), equivalent to *c*. 4 years of global fossil fuel emissions (Boden, Marland, & Andres, 2013) or *c*. 15 years of the global forest carbon sink (Pan et al., 2011). This has led to the incorporation of tree height in REDD+ carbon monitoring (Global Forests Observations Initiative, 2013). Improved plot‐level knowledge of height–diameter relationships would also help improve remote sensing‐based estimates of local and global forest biomass. For example space‐ and airborne LIDAR measure canopy height (Baccini et al., 2012; Saatchi et al., 2011) and high‐quality ground estimates of AGB are needed to calibrate height–AGB allometries (Jucker et al., 2017).

Despite the importance of tree height for estimating biomass, measured heights are frequently unavailable. This has led to the development both of allometric models to estimate AGB without a height parameter (Chave et al., 2005), and of pan‐tropical height–diameter models (Brown, Gillespie, & Lugo, 1989) which are used to predict tree heights when measured heights are unavailable. While these earlier efforts assume that a single height–diameter relationship can be applied across the tropics, height–diameter relationships are known to be influenced by biogeography and by environmental and compositional variation at much smaller scales (Banin et al., 2012; Djomo et al., 2016; Feldpausch et al., 2011; Thomas, Martin, & Mycroft, 2015). Pan‐tropical allometries have therefore been refined to incorporate variation attributed to region (Feldpausch et al., 2012) or climate (Chave et al., 2014). Nevertheless, height–diameter relationships can be expected to vary at all scales, suggesting that even these regionally or climatically modified models themselves lack the necessary sophistication needed for many applications (Rutishauser et al., 2013; Stas, Rutishauser, Chave, Anten, & Laumonier, 2017). It is of course also possible to construct locally derived height–diameter allometries that implicitly incorporate variation due to geography and the environment. Incorporating heights estimated by locally derived models has, for example already been found to reduce estimates of AGB in Central Africa (Kearsley et al., 2013) and to increase estimates of biomass production in Borneo (Banin et al., 2014) when compared with estimates derived from coarser‐scale allometries. Widespread application of locally derived allometries could in principle lead to substantially changed—and improved—estimation and understanding of variation in tropical forest carbon storage and sequestration.

Measuring tree heights is time consuming, so it is rare to measure the heights of all trees within inventory plots. As a result, in practice, local height–diameter relationships are frequently modelled using small samples of trees. For example the RAINFOR field manual recommends measuring the height of 40 trees in 1‐ha plots for convenience where time constraints prevent all trees being measured (Phillips, Baker, Feldpausch, & Brienen, 2009), typically leaving more than 90% of tree heights to be predicted. Height–diameter models parameterised using such small samples of trees may perform poorly at predicting the height of the unmeasured trees, compared to regionally parameterised models using much larger samples of trees, for several reasons: (1) the full range of local diameters may not be sampled, meaning that locally derived models extrapolate beyond the range of data used to train them (see Elith & Leathwick, 2009 for discussion of consequences); (2) non‐linear relationships, such as asymptotic maximum heights, may not be evident within smaller sets of training data (Duncanson, Rourke, & Dubayah, 2015) and (3), models may be excessively influenced by outliers (i.e. trees that are unusually tall or short for their diameter). It is thus uncertain how many trees need to be sampled to ensure that locally derived models constructed using small samples of trees actually do yield better estimates of tree height than regional models. Furthermore, it would be very helpful to understand, generally, how sampling effort in the field impacts the reliability of local‐scale models across tropical forests. In particular, ecologists and practitioners aiming to generate improved accuracy of forest biomass estimates would benefit from knowing the sample size(s) and sampling protocols required to ensure that locally derived models consistently outperform existing regional and climate‐based models.

Here we addressed these challenges by assembling a pan‐tropical dataset of plots where large numbers (≥150 per plot) of trees have been sampled for height measurement and examining these to quantify how well locally derived models predict tree height. We use a cross‐validation approach to allow us to test height–diameter model performance on data that are independent to those used for model fitting. Our specific objectives were to (1) examine how the number of trees used to train locally derived models affects prediction errors with reference to the performance of existing regional and climate‐based models and (2) test different strategies for sampling trees to produce locally derived models.

## 2 MATERIALS AND METHODS

### 2.1 Forest inventory data

Pan‐tropical inventory data were collected by three networks of ecologists, working in South America (RAINFOR, Malhi et al., 2002), Africa (AfriTRON, Lewis et al., 2013) and Southeast Asia (T‐FORCES, Qie et al., 2017), with all following standardised protocols that include diameter measurement of all trees ≥10 cm *D* measured at 1.3 m or above buttresses. Data were curated in the ForestPlots.net database (Lopez‐Gonzalez, Lewis, Burkitt, & Phillips, 2011), and subject to identical quality control and quality assurance procedures. From this dataset we selected plots in intact, lowland (<1,500 m a.s.l.) closed canopy forest. Annual precipitation, obtained from the WorldClim database (Hijmans, Cameron, Parra, Jones, & Jarvis, 2005), ranged from 1,339 to 3,806 mm, whereas mean annual temperatures were between 22.6°C and 27.1°C. We selected plots where at least 150 trees had height measurements that met the criteria for inclusion (*n* = 53 plots) or where combinations of plots within 5 km of each other with comparable forest composition, elevation and edaphic conditions had ≥150 trees with height measurements (*n* = 96 individual plots combined into 20 plots, hereafter also referred to as “plots”). The criteria for including individual height measurements were (1) tree stems were not broken, leaning by ≥10% or fallen, (2) tree heights were measured either using clinometers, laser rangefinders, laser hypsometers or directly by climbing and (3) tree heights were below 90 m (heights above this were assumed to be errors). Following application of these filters, our dataset consisted of 73 plots (30 in South America, 30 in Africa and 13 in Asia) with 28,173 trees with measured heights.

### 2.2 Height–diameter models

We used three equations to relate measured heights (*H*) to tree diameters (*D*) in each plot (subsequently referred to as locally derived models). First, we used the Weibull function

(1) $$H=a\left(1-\exp\left(-bD^{C}\right)\right),$$

where *a*,* b* and *c* are estimated parameters. An intuitive property of the Weibull function is that *a* can be interpreted as the asymptotic maximum height of a tree.

Second, we used the Michaelis–Menten function

(2) $$H=\left(\mathrm{aD}\right)/\left(b+D\right),$$

where *a* and *b* are estimated parameters. For both Weibull and Michaelis–Menten models, we also fitted height–diameter models with case weights proportional to the volume of each tree (Molto et al., 2014). These weights give more importance to large trees during model fitting, and may improve estimates of stand‐level AGB as these large trees are dominant components of stand‐level biomass due to the nonlinear relationship between *D* and AGB (Bastin et al., 2015; Slik et al., 2013).

Third, we modelled the height–diameter relationship using the log–log linear ordinary least squares regression

(3) $$\ln\;\left(H\right)=a+b\left(\ln\left(D\right)\right),$$

where *b* gives the scaling exponent of a power law relationship between height and diameter.

Height–diameter models were fitted in R (R Core Team, 2014) using functions in the biomass r package (Réjou‐Méchain, Tanguy, Piponiot, Chave, & Hérault, 2017), with the nonlinear Weibull and Michaelis–Menten models parameterised using the Levenberg–Marquard algorithm implemented in the minpack.lm r package (Elzhov, Mullen, Spiess, Bolker, & Mullen, 2016). All five models were parameterised separately for each set of training data in each plot.

We compared these locally derived models to regionally parameterised height–diameter Weibull equations (i.e. same form as Equation (1)) with parameters for each biogeographical region obtained from Feldpausch et al. (2012), and to the pan‐tropical climate‐based model

(4a) $$\ln\;\left(H\right)=-0.893-E\;+\;0.760\;\ln\;\left(D\right)-0.0340{\left[\ln\;\left(D\right)\right]}^{2}$$

from Chave et al. (2014), where *E* is defined as

(4b) $$E=\left(0.178\;\times \;T-0.938\;\times \;C-6.61\;\times \;P\right)\;\times \;10^{-3}$$

*C* is climatological water deficit, *T* is temperature seasonality and *P* is precipitation seasonality, see Chave et al. (2014) for further details.

### 2.3 Evaluating model performance

The performance of height–diameter models was assessed by training models on a subset of trees within a plot, before randomly selecting 50 of the remaining trees and predicting the height of these. Prediction error was calculated as the square‐root of the mean squared difference between measured and predicted heights (i.e. root‐mean squared error, RMSE). This approach allows the performance of models to be assessed on independent testing data. We note that while we define prediction errors as differences between predicted and measured heights, the measurement of tree height itself is also subject to errors (see Larjavaara & Muller‐Landau, 2013). Reported differences between measurement instruments did not affect our results as inferences about the performance of locally derived allometries were not affected by restricting analyses to measurements made with clinometers (Figure S1).

We first assessed whether locally derived models had lower prediction errors than regional models by splitting data for each plot into independent training and testing subsets, fitting Weibull, Michaelis–Menten and log–log height–diameter models to the training subset and calculating the prediction errors of both these locally derived models, and regional and climate‐based models, on the testing subset. We did this for training data sample sizes of 10 up to 100 trees, in increments of 10 trees. For a given sample size, we randomly selected training and testing subsets for 100 iterations. We used linear mixed‐effects models to quantify the difference in prediction error among height–diameter models, with plot identity and sample identity (i.e. an identifier for each division of the data into training and testing subsets) as random effects; 95% confidence intervals were obtained by parametric bootstrap. We fitted separate mixed‐effects models to sample size increments of 10 and 100 trees. For each height–diameter model, we also modelled the probability of it being the best performing model in a given sample of trees as a function of training data sample size using generalised linear mixed effects models with binomial errors and a logit link, with plot identity as a random effect.

To provide an objective measure of any turning points in the relationship between RMSE and sample size, and hence evaluate whether there are any threshold sample sizes beyond which further sampling gives diminishing returns, we numerically estimated the second derivative (Fewster, Buckland, Siriwardena, Baillie, & Wilson, 2000) of the smoothed relationship between RMSE and sample size as

(5) $$\text{Second derivative}={I_{n}}_{+2}-2I_{n}+{I_{n}}_{-2}/4,$$

where *I*_*n*_ is the trend curve at sample size *n*. We expected the relationship between RMSE and sample size to be negative, with potentially saturating rates of decline. For negative relationships, positive second derivative values indicate a slowing in the rate of change, so that peaks in the second derivative highlight threshold sample sizes beyond which returns from further sampling diminish. The trend curve was obtained by fitting a generalised additive model, implemented in the mgcv r package (Wood, 2006), of RMSE as a function of sample size, setting the maximum base dimension of the spline to four. The exact turning point is sensitive to the degree of smoothing of the trend curve, so we interpret results from this method alongside visual inspection of relationships.

To evaluate how height prediction errors propagated to errors in AGB estimates, we used the allometric equation of Chave et al. (2014), implemented in the biomasaFP r package (Lopez‐Gonzalez, Sullivan, & Baker, 2015), to estimate the AGB of each tree from their diameter *D* and estimated height *H*

(6) $${\text{AGB}}_{\mathrm{est}}\;=\;0.0673\;\times {\left(\rho D^{2}H\right)}^{0.976},$$

where ρ is wood density derived from Chave et al. (2009) and Zanne et al. (2009). Although we do not know the true AGB of trees in our dataset, as trees were not destructively sampled, we can identify errors in AGB estimates due to the height component of allometric equations by comparing estimates of AGB using observed heights with estimates using modelled heights. We therefore used the difference between the summed AGB of the 50 trees in the testing dataset when height was predicted using a height–diameter model and when observed height was used as an indication of stand‐level AGB prediction errors.

### 2.4 Evaluating different strategies for sampling trees for height measurement

To evaluate whether different strategies for sampling trees reduced height prediction errors we evaluated prediction errors of locally derived Weibull and Michaelis–Menten models (selected as these were the best performing models, see Results) trained using samples of trees selected using different sampling strategies. These were (1) randomly sampling *n* trees (Rand), (2) sampling *n* trees in proportion to the number of trees in different size classes (<200 mm *D*, ≥200 mm *D* and <300 mm *D*, ≥300 mm *D* and <500 mm *D* and ≥500 mm *D,* Strat), (3) sampling the *n* trees with the largest diameter (Big), (4) sampling the 10 largest trees then randomly sampling the remaining *n*−10 trees (BigRand) and (5) sampling the 10 largest trees and taking a size‐class stratified random sample of the remaining *n*−10 trees (BigStrat). We repeated this for samples of 10 to 100 trees in increments of 10, and took 100 samples from each plot and each sample size. Some sampling strategies (e.g. sampling the *n* largest trees) systematically removed a portion of trees from the testing dataset, so differences between sampling strategies evaluated using independent testing data may arise through differences in the variance of tree heights in the testing dataset. To avoid this, we tested model performance using all trees with a height measurement in the plot in this analysis. We then calculated mean RMSE and stand‐level AGB prediction errors for each sample size and plot, and for each plot‐sample size combination identified which sampling strategy gave the smallest RMSE and minimum absolute AGB prediction error (identified as the lowest prediction error across the Weibull and Michaelis–Menten models). The probability of a sampling strategy resulting in the best performing model was then modelled for both height RMSE and AGB prediction error as a function of sample size using generalised additive models, setting the maximum base dimension of the spline to four as a compromise between allowing nonlinear relationships and avoiding overfitting.

## 3 RESULTS

### 3.1 Performance of locally derived models

**Figure 1:**
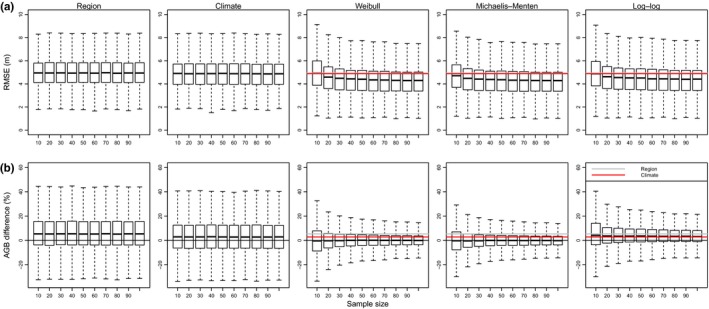
Relationship between the number of height measurements used to train tropical tree height–diameter models and (a) height prediction error (the square‐root of mean squared error, RMSE) when tested on an independent sample of 50 trees in the same permanent sampling plot and (b) difference in the above‐ground biomass (AGB) of these 50 trees when estimated using predicted height and when estimated using observed height. The performances of regional (Feldpausch et al., 2012) and climate‐based (Chave et al., 2014) height–diameter models tested on the same testing data are shown for comparison with locally derived Weibull, Michaelis–Menten and log–log models. Boxplots show variation in values averaged across iterations for each sample size in each plot. For clarity, outliers (points >1.5 × box length away from the upper or lower quartile) are not plotted. The grey line in each plot shows the median RMSE value for regional height–diameter models pooled across all plots and iterations, whereas the red line shows the median RMSE value for the climate‐based height–diameter model—in some cases only one line is visible due to over‐plotting

On average, locally derived height–diameter models predicted the height of independent samples of trees more accurately than biogeographical region or climate‐based models (Figure 1a). When only 10 height measurements were used to train models, height prediction errors of Michaelis–Menten models were statistically significantly lower than those of regional models obtained from Feldpausch et al. (2012) (statistical significance indicated by confidence intervals of difference in prediction error from regional models not overlapping zero) or a climate‐based model obtained from Chave et al. (2014), with a reduction in prediction error from regional models of 0.18 m (95% CI = 0.15–0.21 m). When 20 height measurements were used to train models, all locally derived model forms had lower prediction errors than regional or climate‐based models. Reductions in prediction error were greatest for Michaelis–Menten models (mean difference = −0.46 m, 95% CI = −0.44 to −0.48 m), followed by Weibull models (mean difference in prediction error from regional model = −0.35 m, 95% CI = −0.33 to −0.37 m) then log–log models (mean difference = −0.31 m, 95% CI = −0.29 to −0.33 m). The prediction errors of local height–diameter models decreased with increasing sample size (Figure 1a), and were >0.5 m lower than those of regional models when 100 height measurements were used to train local models (Weibull model: mean difference = −0.67 m, 95% CI = −0.66 to −0.69 m; Michaelis–Menten model: mean difference = −0.68 m, 95% CI = −0.66 to −0.69 m; log–log model: mean difference = −0.56 m, 95% CI = −0.55 to −0.58 m). Weighted forms of Weibull and Michaelis–Menten models showed smaller improvements in prediction error (weighted Weibull: mean difference from regional model = −0.48 m, 95% CI = −0.47 to −0.50 m, weighted Michaelis–Menten model: mean difference = −0.43 m, 95% CI = −0.42 to −0.45 m). Prediction errors were significantly lower when climate‐based height–diameter models were used than when regional models were used (mean difference = −0.09 m, 95% CI = −0.07 to −0.11 m), although there was considerable variation in the performance of these two methods among plots (Figure 1a).

**Figure 2:**
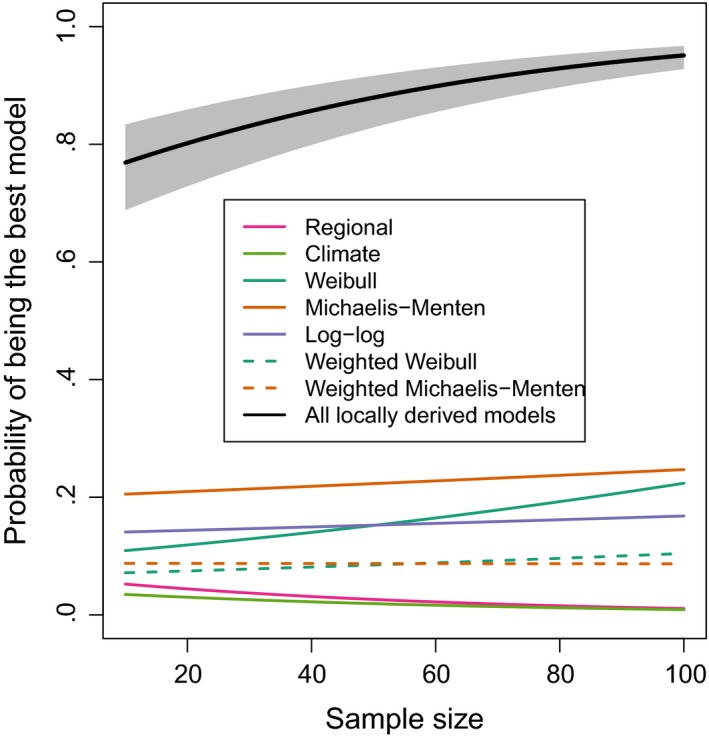
Relationship between sample size and the probability of a given height–diameter model being the best performing model for a sample of tropical trees. The probability of a given height–diameter model being the best performing model was modelled as a function of sample size using generalised linear mixed effects models with binomial errors and a logit link, with plot identity as a random effect. We also modelled the probability that one of the five local height–diameter models was the best performing model (Local). For the latter, fitted relationships and 95% confidence intervals are shown. 95% confidence intervals where calculated based on a normal approximation on the scale of the linear predictor. Note that for a sample size of 40 trees local height–diameter models are six times more likely to provide a better fit than either a biogeographical regional or climate model

The lower mean prediction error of local models was reflected in the high probability of a local model being the best height‐diameter model for a sample of trees (Figure 2). When local models were trained on samples of 10 trees, the probability of the model with the lowest height prediction errors being one of the five locally derived models was 0.77 (95% CI = 0.69–0.83), rising to 0.86 (95% CI = 0.80–0.90) when 40 trees were sampled and 0.95 (95% CI = 0.93–0.97) when 100 trees were sampled. Note that this analysis includes occasions when nonlinear models did not to converge as failures, so the superior performance of locally derived models is robust to convergence failure. No single locally derived model consistently outperformed the others (Figure 2), although at small sample sizes Michaelis–Menten models outperformed other models (probability of being best model when 10 trees were sampled = 0.21, 95% CI = 0.18–0.23, cf. Weibull 0.11, 95% CI = 0.10–0.12). However, when all trees in a plot were used to construct allometric models, Weibull models had the lowest height RMSE in 92% of plots, Michaelis–Menten in 7% and log–log in 1% (Figure S2).

Locally derived Weibull and Michaelis–Menten height–diameter models provided unbiased estimates of stand‐level biomass (stand‐level biomass defined here as AGB summed over the 50 trees in the training dataset) relative to estimates using observed height, and also had lower AGB prediction errors than regional and climate‐based models (Figure 1b). In contrast, log–log models showed a tendency to overestimate stand‐level biomass relative to estimates using observed height (Figure 1b).

### 3.2 Effect of sample size

There were diminishing returns in improvement in model performance with increasing sample size (Figure 1). For Weibull models and log–log, the greatest decrease in the gradient of the fitted generalised additive model of the relationship between height prediction error and sample size (as indicated by the maximum value of the second derivative) occurred once 40 height measurements were used, whereas for Michaelis–Menten models this occurred when 41 trees were sampled. Visual inspection of relationships support this (Figure 1a) and indicate that similar flattening occurred for the probability of a locally derived model outperforming a regional model (Figure 2) when 30–50 height measurements were used.

### 3.3 Evaluation of different sampling strategies

**Figure 3:**
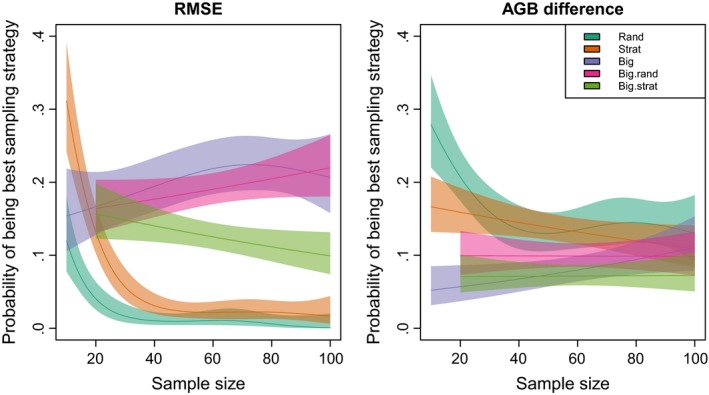
Probability of different sampling strategies resulting in the best performing tropical tree local height–diameter model, where model performance was assessed as (1) height root‐mean squared error (RMSE) and (2) the difference between estimated stand‐level above‐ground biomass (AGB) using modelled heights and stand‐level above‐ground biomass (AGB) estimated using observed heights. *n* trees were sampled either randomly, stratified according to size class (Strat), the largest *n* trees were sampled (Big), the 10 largest trees where sampled with the remaining trees randomly sampled or stratified by size class (BigStrat). For each plot. The probability of a sampling strategy resulting in the best performing model was modelled as a function of sample size using generalised additive models. Fitted relationships and 95% confidence intervals are shown

For samples sizes of greater than 20, sampling strategies that included the 10 trees with the largest diameter had a statistically significantly higher probability of resulting the model with lowest height prediction error (Figure 3). Although the strategy of sampling the largest *n* trees performed well on average (Figure 3), for some plots it resulted in very high prediction error (Figure S3). Random and size class stratified sampling strategies were more likely to produce models that minimised AGB prediction error, although there was considerable overlap in confidence intervals at larger sample sizes (Figure 3). Note that for both height and AGB prediction error, the probability of a given sampling strategy producing the best model was low (<0.3), indicating that no single sampling strategy consistently outperformed the others.

## 4 DISCUSSION

Although the importance of tree height in allometric models used to estimate tropical tree biomass is widely recognised (Feldpausch et al., 2012), it is rare to measure the heights of all trees in a permanent sample plot, meaning that it is often necessary to use existing allometric models to estimate tree height (Chave et al., 2014; Feldpausch et al., 2012). Our results show that sampling as few as ten trees in a plot is, on average, sufficient to construct height–diameter allometries that perform better than existing regional or climate‐based models. Sampling further trees improved locally derived allometries, albeit with diminishing returns. Analysis of turning points supports the use of a threshold of 40 trees as a compromise between fieldwork effort and improvements in model performance. Our results demonstrate that with remarkably limited fieldwork effort it is possible to collect local height data that will improve estimates of forest biomass. More widespread collection of height data will of course also be useful to further understanding of spatial variation in forest architecture (Banin et al., 2012; Chave et al., 2014; Feldpausch et al., 2011) and to further develop regional and pan‐tropical height–diameter allometries.

While our results demonstrate the potential for local height–diameter allometries to refine understanding of spatial variation in carbon stocks, the consequences of using local height‐diameter allometries for estimates of total carbon stocks in tropical forests are unclear, with regional models tending to overestimate tree height in some areas and underestimate it in others. For example in Central Africa, estimates of carbon stocks were reduced when local height–diameter allometries were used instead of regional models (Kearsley et al., 2013), whereas in Borneo the use of local height‐diameter allometries increased estimates of above‐ground woody production compared to estimates using pan‐tropical allometries (Banin et al., 2014). In our pan‐tropical dataset, climate‐based and regional height‐diameter allometries tended to slightly overestimate stand‐level AGB relative to estimates using observed height, but this effect varied considerably among plots (Figure 1b).

Despite the reduction in height prediction error when locally derived allometries were used, prediction errors of around 4 m remained even when using locally derived allometries (Figure 1). Substantial variability around average relationships persisted when all trees in a plot were used to construct allometries (Figure S2), and may be due to species‐specific differences in allometry (Goussanou et al., 2016) or variation in the local competitive environment within stands (Forrester, Benneter, Bouriaud, & Bauhus, 2017). A potential source of within‐plot variation is topography. This can influence height‐diameter relationships, with taller canopies in valleys than ridges (Detto, Muller‐Landau, Mascaro, & Asner, 2013). Because of this, the performance of height‐diameter allometries in topographically heterogeneous plots may be improved by stratifying sampling by topography. Available data suggest variation in tree height may be greatest at scales >100 m (Detto et al., 2013) so this may become an important consideration in plots considerably larger than 1 ha.

Our analysis focuses on the consequences of different sample sizes and strategies for the performance of height‐diameter models, so prediction errors result from the fit of statistical models. However, it is important to note that the measurement of tree height itself is also subject to random and directional error. We anticipate that the latter will have the greatest consequences for the construction of regional and local height‐diameter allometries, leading to models systematically under or over‐predicting tree height and hence biomass. To date there have been few attempts to quantify the magnitude of such errors (Larjavaara & Muller‐Landau, 2013 is a notable exception). We reiterate Larjavaara & Muller‐Landau's call for more studies to tackle this issue—better understanding is needed of how measurement errors vary among biogeographical regions, across environmental gradients, with forest structure and with human and technical factors in order to develop appropriate correction factors and to understand their impact on tropical forest biomass estimates.

The performance of locally derived height‐diameter models was influenced by the form of the allometric equation used. We used three‐parameter Weibull, log–log linear and Michaelis–Menten models relate tree height to tree diameter, but alternative model structures (e.g. Gompertz) could have been used (Ledo et al., 2016). As our aim was to investigate the consequences of in‐field sampling decisions rather than post‐fieldwork modelling choices, we did not explore the full range of possible models. However, a previous evaluation of 12 allometric models recommended using three‐parameter Weibull models (Ledo et al., 2016). Our results are somewhat consistent with this, as although log–log models were sometimes the best performing model when samples sizes were small, prediction errors of Weibull models were on average lower than those of log–log models at all sample sizes. However, we also found that Michaelis–Menten models performed better on average than Weibull models (in terms of reducing height prediction error) when sample sizes were small, with the relative performance of Weibull models increasing with sample size. The failure of a single model form to consistently outperform others at minimising height prediction errors (Figure 2) supports previous studies that have found that the best performing model form varies between forest types (Cuni‐Sanchez et al., 2017). For example in locations with frequent natural disturbances trees may not reach their asymptotic maximum heights, and in these plots log–log models may perform better than asymptotic models. Despite this general variability, our results also indicate that log–log models were biased towards overestimating tree height and hence AGB (relative to AGB estimates using observed heights), especially when trained on small sample sizes that were likely to miss the largest diameter trees. This is consistent with a previous investigation of the sample size sensitivity of the power law relationship between crown radius and tree height, which found that power law models overestimated tree height when trained on small samples of trees (Duncanson et al., 2015). In contrast, local Weibull and Michaelis–Menten models showed little bias in stand‐level AGB estimates, even when trained on small samples of trees (Figure 1b), supporting the use of asymptotic models of tree height–diameter relationships (Fayolle et al., 2016; Ledo et al., 2016).

The best sampling strategy differed depending on whether performance was assessed by height prediction errors or AGB prediction errors (Figure 3). This could result from a tension between maximising the fit of height‐diameter models for small trees and maximising fit for large trees, as sampling strategies focused on capturing the height‐diameter relationships of the largest trees performed less well than random sampling at predicting stand‐level AGB, potentially due to overestimation of the heights of smaller trees. We interpret this tension as indicating that while Weibull and Michaelis–Menten height‐diameter relationships give a good approximation of true height‐diameter relationships in most plots, there is insufficient parameterisation to describe the differences in allometries between small and large trees. This is consistent with a previous assessment which found a tendency for Weibull models, along with other three‐parameter asymptotic functions, to underestimate the height of the largest trees (Banin et al., 2012). Differences in allometry between small and large trees could result from differences in the severity of light competition and exposure to high winds between the canopy and understory (O'Brien, Hubbell, Spiro, Condit, & Foster, 1995), and possible hydraulic limitation of large trees (Ryan & Yoder, 1997), and supports the idea that the allometery and abundance of canopy trees may be constrained differently to those of understory trees (Farrior, Bohlman, Hubbell, & Pacala, 2016). It may be desirable to give more weight to errors in the prediction of the heights of large trees than errors for small trees as AGB is nonlinearly related to tree diameter. This can be achieved by applying case weights proportional to tree volume when fitting height‐diameter models. Surprisingly, we found that these weighted models tended to perform worse than unweighted models (Figure 2).

It is important to note that we did not perform an exhaustive comparison of all possible sampling strategies. For example a strategy of sampling all emergent trees would ensure that the tallest trees are measured, so may perform better than strategies based on sampling the trees with the largest diameters.

Although our results show that locally derived height‐diameter models can be constructed with 40 height measurements, there will remain cases where no local height data are available. In these cases, it will be necessary to use height‐diameter models developed at other locations. Pan‐tropical height‐diameter models have been refined to include variation in allometry with climate (Chave et al., 2014) or among biogeographical regions (Feldpausch et al., 2012). Our results still support the use of these models when local height data are not available, as reductions in prediction error with locally derived allometries were, on average, less than 1 m. We show that the relative performance of regional and climate‐based models were similar, with slightly lower prediction errors from the climate‐based model on average, although this varied among plots. However, biogeographical region is known to have a strong influence on tree allometry (Banin et al., 2012), so it is likely that allometric models could be improved by incorporating both variation in climate and region. Furthermore, accounting for local variation in height‐diameter relationship is key in forests that have experienced recurrent climatic (Thomas et al., 2015) or human disturbances (Rutishauser, Hérault, Petronelli, & Sist, 2016), and where generic models developed in more preserved forests are likely to return wrong estimates.

### 4.1 Recommended protocol for sampling trees for height measurement

Measuring more tree heights had diminishing returns in terms of reductions in height prediction error. We found the strongest reduction in the slope of the relationship between sample size and prediction error to be when 41 trees were sampled, but as prediction errors continue to decline with increasing sample size we recommend sampling 50 trees as a conservative threshold. Sampling the largest trees reduced height prediction error, but biomass estimates were more accurate when random or stratified sampling was used. The strategy of sampling the ten largest trees in a plot, then randomly sampling the remaining trees showed intermediate performance in both height and biomass prediction, but stratified sampling of the remaining trees may be more preferable as it ensures height data are available for trees of each size class. Following these recommendations, the procedure in the field would simply be to first identify the ten largest diameter trees in a plot for height measurement, then take a diameter size class stratified random sample of a further 40 trees for careful height measurement.

## AUTHORS’ CONTRIBUTIONS

O.L.P., S.L.L., J.L. and Y.M. conceived the RAINFOR, AfriTRON and T‐FORCES forest census network programmes, M.J.P.S., S.L.L. and O.L.P. conceived and designed this study, O.L.P., S.L.L., T.R.B., W.H., L.Q., L.F.B., A.C.‐S., T.R.F., T.S. and R.J.W.B. coordinated data collection with the help of most co‐authors, G.L.G., O.L.P., S.L.L., T.R.B. contributed tools to analyse and curate data, all authors except M.J.P.S. collected field data, M.J.P.S. analysed the data with input from other co‐authors, M.J.P.S., S.L.L. and O.L.P. wrote the paper. All co‐authors commented on or approved the manuscript.

## DATA ACCESSIBILITY

Data used in this paper are available from https://doi.org/10.5521/forestplots.net/2018_1 (Sullivan et al., 2018).
