# Supplementary material for: Field methods for sampling tree height for tropical forest biomass estimation
Source: Methods Ecol Evol. 2018 Feb 13;9(5):1179–89. doi: 10.1111/2041-210X.12962 (PMC5993227; doi:10.1111/2041-210X.12962)
Supplement: Supplementary file 1 [file MEE3-9-1179-s001.docx]

**Supporting information for “Field methods for sampling tree height for tropical forest biomass estimation”**

Martin J. P. Sullivan ^1^, Simon L. Lewis ^1, 2^, Wannes Hubau ^1, 3^, Lan Qie ^1, 4^, Timothy R. Baker ^1^, Lindsay F. Banin ^5^, Jerôme Chave ^6^, Aida Cuni Sanchez ^2,7^, Ted R. Feldpausch ^8^, Gabriela Lopez-Gonzalez ^1^, Eric Arets ^9^, Peter Ashton ^10^, Jean-François Bastin ^11, 12^, Nicholas J. Berry ^13^, Jan Bogaert ^14^, Rene Boot ^15^, Francis Q. Brearley ^16^, Roel Brienen ^1^, David F. R. P. Burslem ^17^, Charles de Canniere ^18^, Markéta Chudomelová ^19^, Martin Dančák ^20^, Corneille Ewango ^21^, Radim Hédl ^19, 22^, Jon Lloyd ^4^, Jean-Remy Makana ^21^, Yadvinder Malhi ^23^, Beatriz S. Marimon ^24^, Ben Hur Marimon Junior ^24^, Faizah Metali ^25^, Sam Moore ^23^, Laszlo Nagy ^26^, Percy Núñez Vargas ^27^, Colin Pendry ^28^, Hirma Ramírez-Angulo ^29^, Jan Reitsma ^30^, Ervan Rutishauser  ^31, 32^, Kamariah Abu Salim ^25^, Bonaventure Sonké ^33^, Rahayu S. Sukri ^25^, Terry Sunderland ^34^, Martin Svátek ^35^, Peter M. Umunay ^36^, Rodolfo Vasquez Martinez ^37^, Ronald R. E. Vernimmen ^38^, Emilio Vilanova Torre ^29^, Jason Vleminckx ^39^, Vincent Vos ^40, 41^, Oliver L. Phillips ^1^

*^1^ School of Geography, University of Leeds, Leeds LS2 9JT, UK, ^2^ Department of Geography, University College London, London WC1E 6BT, UK, ^3^ Royal Museum for Central Africa, Leuvensesteenweg 13, 3080 Tervuren, Belgium, ^4^ Department of Life Sciences, Imperial College London, Ascot, UK, ^5^ Centre for Ecology and Hydrology, Penicuik, UK, ^6^ Université Paul Sabatier CNRS, UMR 5174 Evolution et Diversité Biologique, Bâtiment 4R1, Toulouse 31062, France, ^7^ Environment Department, University of York, Heslington, York YO10 5NG, ^8^ Geography, College of Life and Environmental Sciences, University of Exeter, Exeter, EX4 4RJ, UK, ^9^ Wageningen Envrionmental Research (Alterra), Wageningen University and Research, PO Box 47, 6700 AA Wageningen, The Netherlands, ^10^ Department of Organismic and Evolutionary Biology, Harvard University, Cambridge, MA, USA, ^11^ UMR AMAP, IRD, Montpellier, France, ^12^ UPR BSEF, CIRAD, Montpellier, France, ^13^ The Landscapes and Livelihoods Partnership, Edinburgh, UK, ^14^ Biodiversity and Landscape Unit, Gembloux Agro-Bio Tech, Université de Liège, Gembloux, Belgium, ^15^ Tropenbos International, PO Box 232, 6700 AE Wageningen, The Netherlands, ^16^ School of Science and the Environment, Manchester Metropolitan University, Manchester, UK, ^17^ School of Biological Sciences, University of Aberdeen, Aberdeen, UK, ^18^ Landscape Ecology and Vegetal Production Systems Unit, Universite Libre de Bruxelles, Brussels, Belgium, ^19^ Institute of Botany, The Czech Academy of Sciences, Brno, Czech Republic, ^20^ Department of Ecology & Environmental Sciences, Faculty of Science, Palacký University, Olomouc, Czech Republic, ^21^ Faculty of Science, Université de Kisangani, Kisangani, Democratic Republic of Congo,  ^22^ Department of Botany, Faculty of Science, Palacký University in Olomouc, Czech Republic****,***  *^23^ Environmental Change Institute, School of Geography and the Environment, University of Oxford, Oxford OX1 3QY, UK, ^24^ Universidade do Estado de Mato Grosso, Campus de Nova Xavantina, Caixa Postal 08, CEP 78.690-000, Nova Xavantina, MT, Brazil, ^25^ Environmental and Life Sciences Programme, Faculty of Science, Universiti Brunei Darussalam, Jln Tungku Link, BE 1410, Brunei Darussalam, ^26^ Universidade Estadual de Campinas, Campinas, Brazil, ^27^ Universidad Nacional de San Antonio Abad del Cusco, Cusco, Perú, ^28^ Royal Botanic Garden Edinburgh, Edinburgh, UK, ^29^ Instituto de Investigaciones para el Desarrollo Forestal, Universidad de Los Andes, Avenida Principal Chorros de Milla, Campus Universitario Forestal, Edificio Principal, Mérida, Venezuela, ^30^ Bureau Waardenburg bv, Postbus 365, Culemborg AJ 4100, The Netherlands, ^31^ Carboforexpert, Geneva, Switzerland, ^32^ Smithsonian Tropical Research Institute, Box 0843-03092, Balboa, Ancon, Panama, ^33^ Plant Systematic and Ecology Laboratory, Department of Biology, Higher Teachers’ Training College, University of Yaounde I, PO Box 047, Yaounde, Cameroon, ^34^ Center for International Forestry Research, Bogor, Indonesia, ^35^ Mendel University in Brno, Department of Forest Botany, Dendrology and Geobiocoenology, Faculty of Forestry and Wood Technology, Brno, Czech Republic, ^36^ Yale School of Forestry & Environmental Studies, 195 Prospect Street, New Haven, CT 06511, USA, ^37^Jardín Botánico de Missouri, Prolongacion Bolognesi Mz.e, Lote 6, Oxapampa, Pasco, Perú, ^38^ Deltares, Delft, The Netherlands, ^39^ Service d’Évolution Biologique et Écologie, CP160/12, Université Libre deBruxelles, 50 av. F. D. Roosevelt, 1050 Brussels, Belgium, ^40^ Universidad Autónoma del Beni, Campus Universitario, Av. Ejército Nacional, Riberalta, ^41^Bolivia Centro de Investigación y Promoción del Campesinado - Norte Amazónico, Cl. Nicanor Gonzalo Salvatierra N° 362, Riberalta, Bolivia*


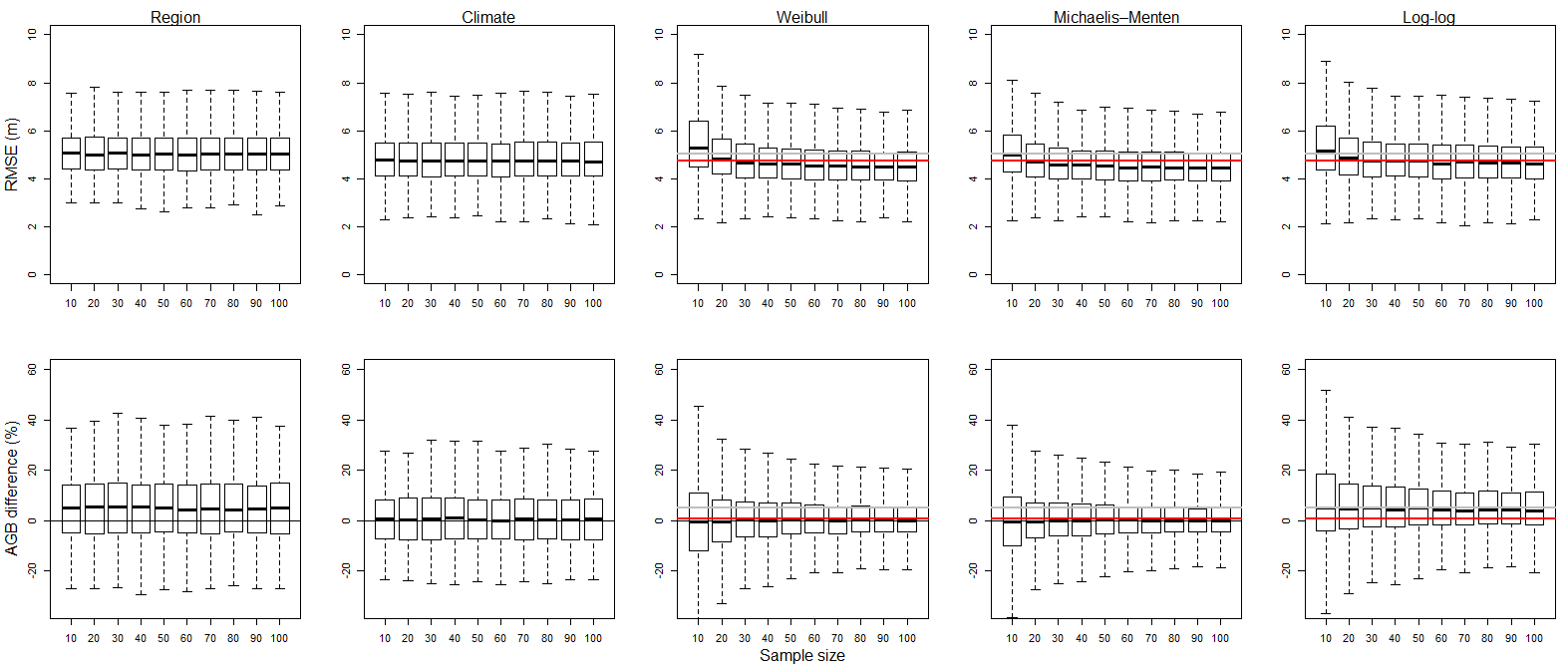


**Figure S1.** As Figure 1, but excluding laser rangefinder height measurements, and thus exploring the sensitivity of our results to potential bias in laser rangefinder measurements relative to clinometer measurements. Only plots where no trees were measured with laser rangefinders are included (n = 16). The red line shows the median RMSE/ AGB difference when tree heights were predicted based on relationships with climate, and the grey line shows median prediction error from when tree heights were predicted from a regional height-diameter model. Note that local Weibull and Michaelis-Menten height-diameter models have, on average, lower prediction errors than regional or climate based models when samples sizes of more than 20 trees are used.


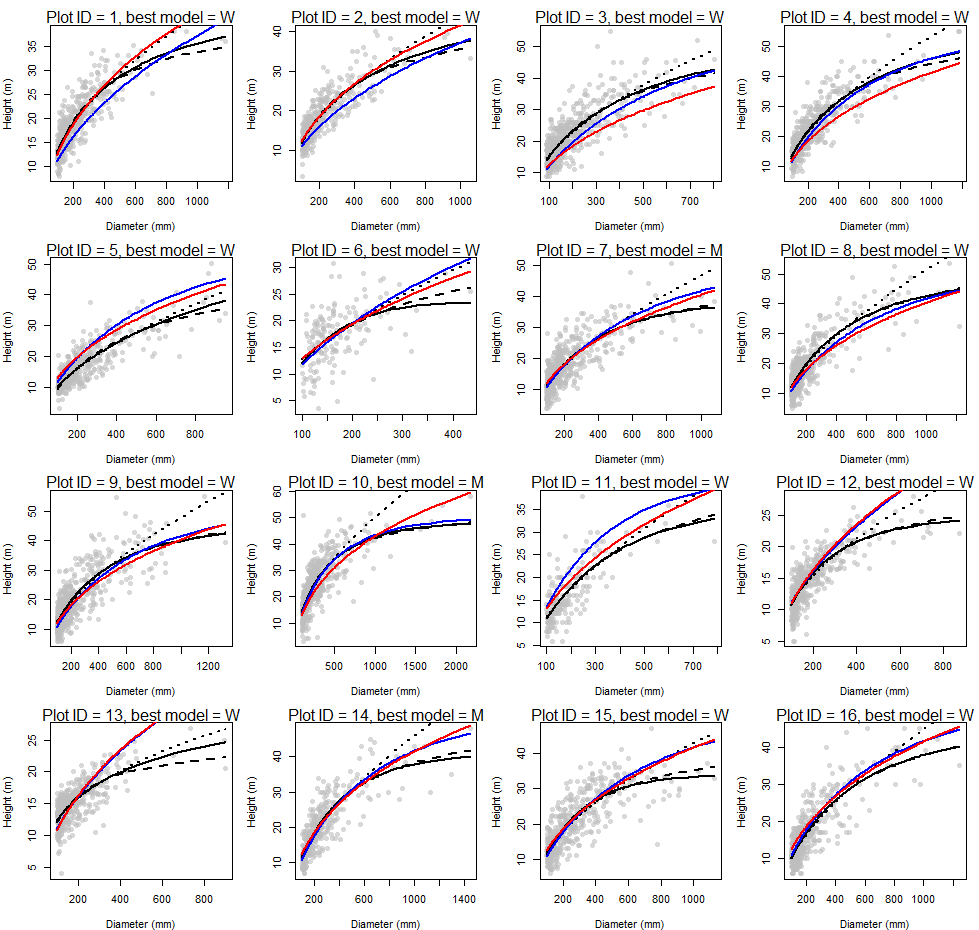


**Figure S2.** Height-diameter relationships in each plot. Fits of locally derived models are shown in black (Weibull model = solid line, Michaelis-Menten model = dashed line, log-log model = dotted line), the regional model shown in blue, and the climate based model shown in red. The plot ID and model with the lowest height RMSE is shown above each graph. W = Weibull, M = Michaelis-Menten, L = log-log, C = climate, R regional. Figure continued overleaf.


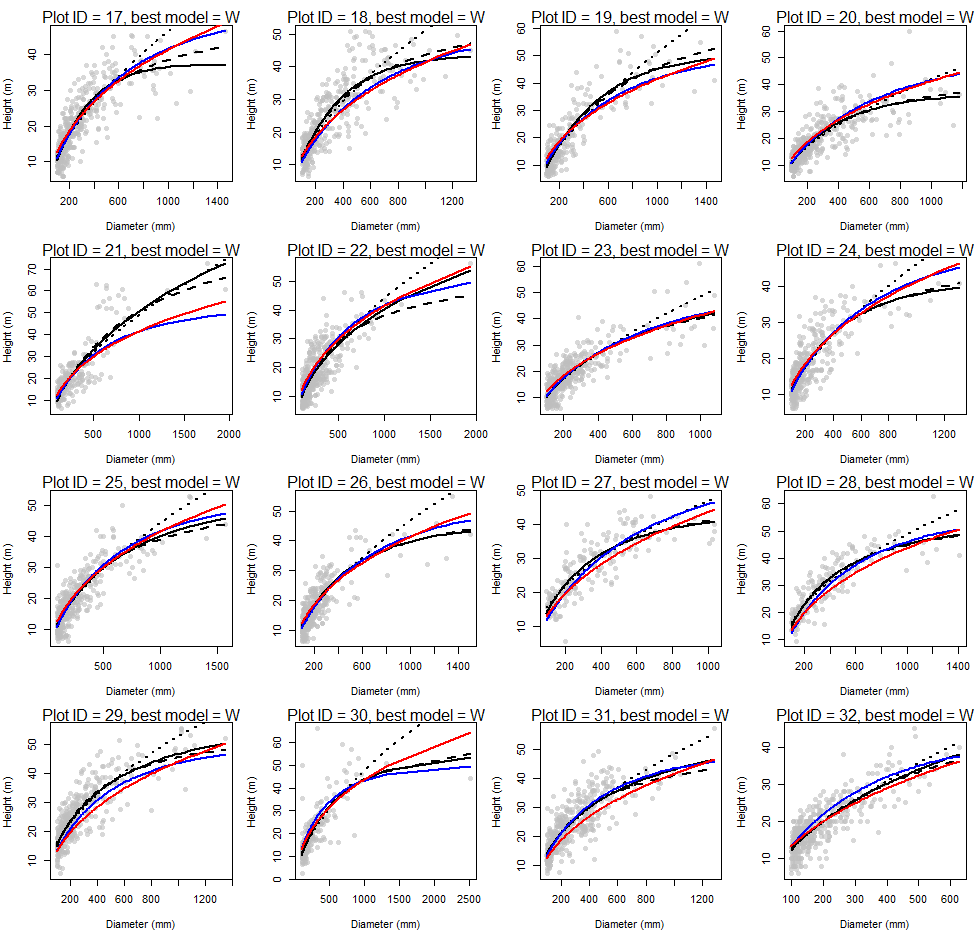


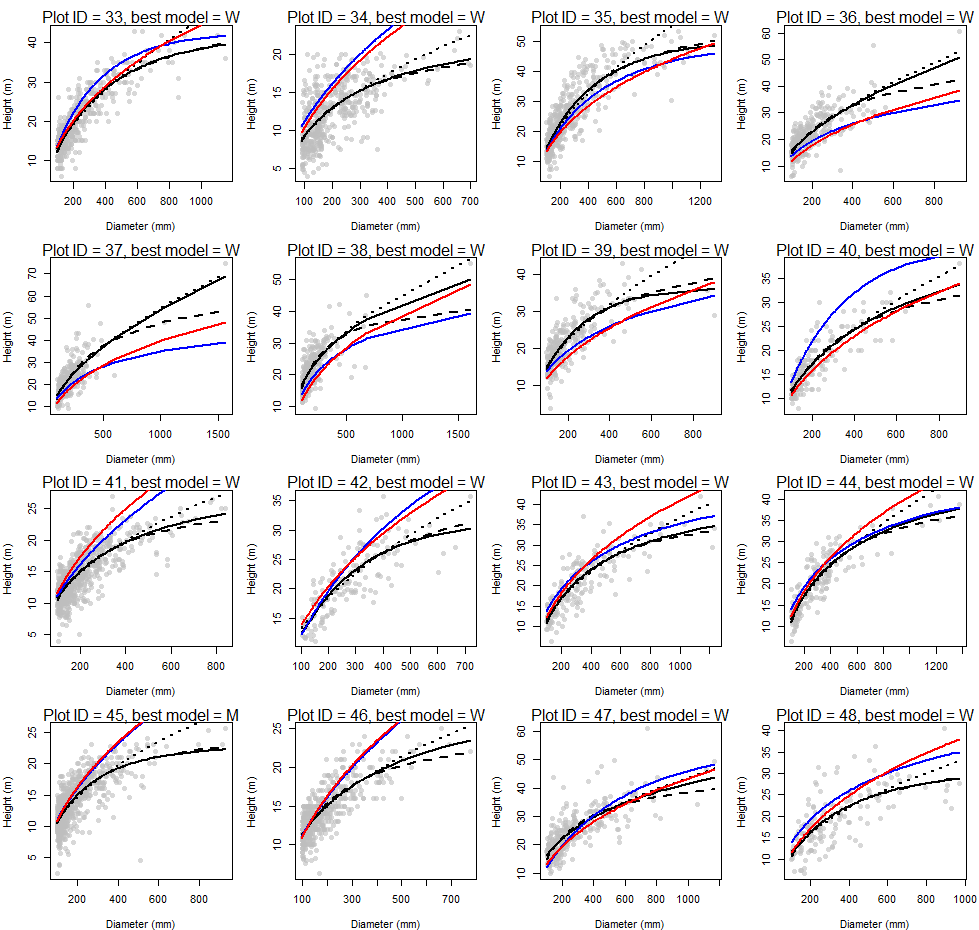


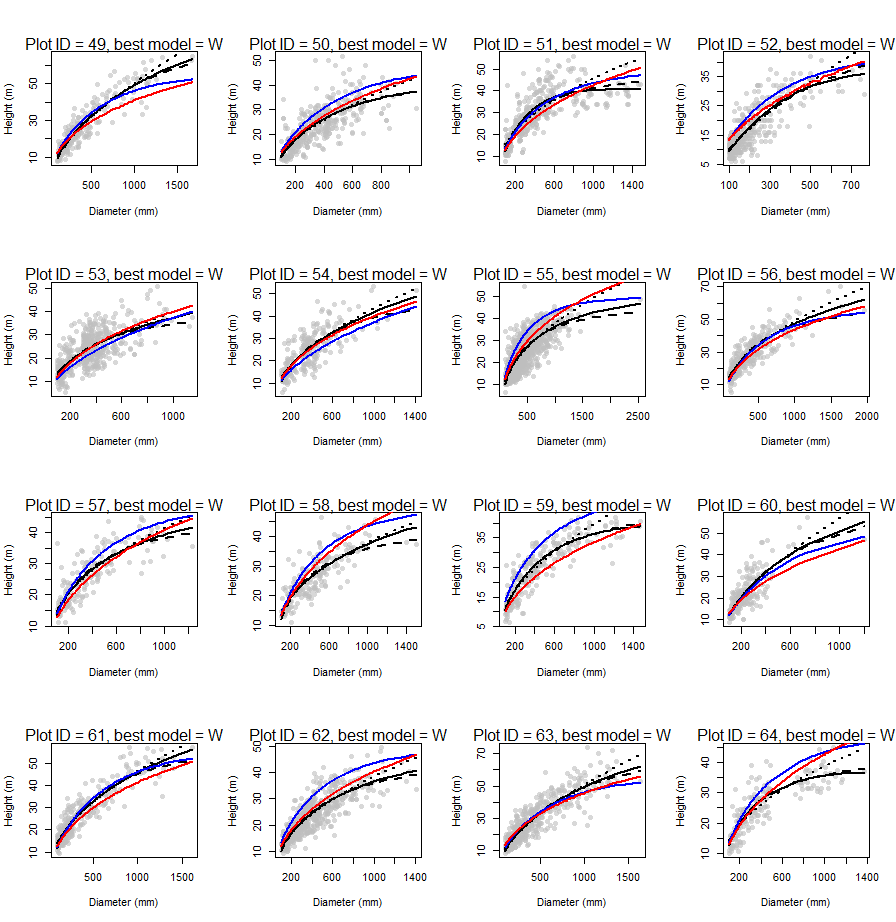


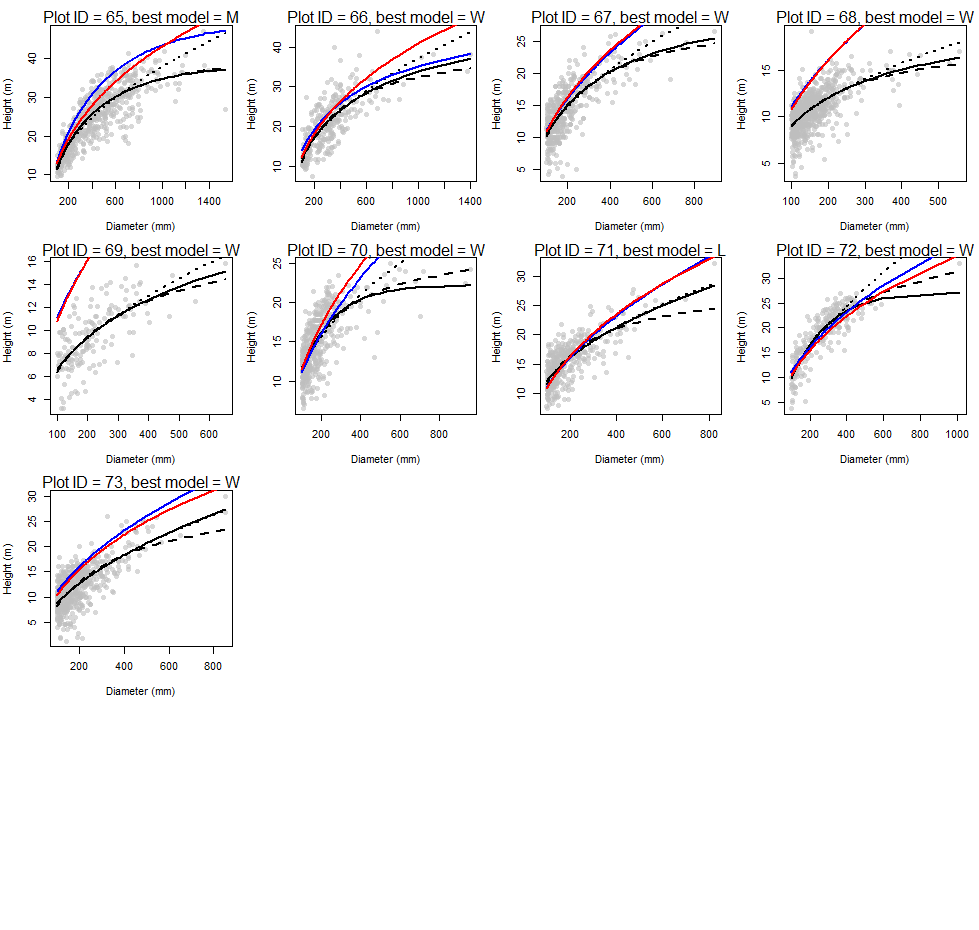


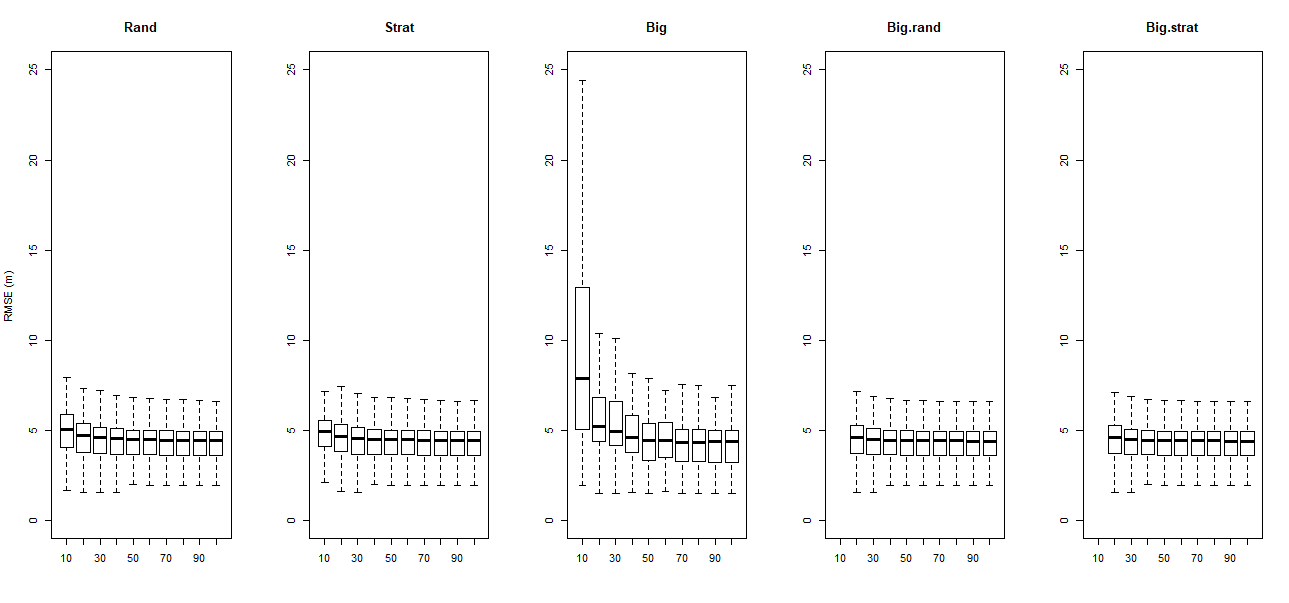


Figure S3. Variation in height prediction error among plots when different sampling strategies are used. Rand = random sample, Strat = stratified sample, Big = sample the *n* largest trees, Big rand = sample the 10 biggest trees then the remainder randomly, Big.strat = sample the 10 biggest trees then the remainder stratified by size class.
